# Prosody Predicts Contest Outcome in Non-Verbal Dialogs

**DOI:** 10.1371/journal.pone.0166953

**Published:** 2016-12-01

**Authors:** Amélie N. Dreiss, Philippe G. Chatelain, Alexandre Roulin, Heinz Richner

**Affiliations:** 1 Department of Ecology and Evolution, University of Lausanne, Lausanne, Switzerland; 2 Institute of Ecology and Evolution, University of Bern, Bern, Switzerland; 3 Center for Plant Molecular Biology, University of Tübingen, Tübingen, Germany; IRCCS Istituto Auxologico Italiano, ITALY

## Abstract

Non-verbal communication has important implications for inter-individual relationships and negotiation success. However, to what extent humans can spontaneously use rhythm and prosody as a sole communication tool is largely unknown. We analysed human ability to resolve a conflict without verbal dialogs, independently of semantics. We invited pairs of subjects to communicate non-verbally using whistle sounds. Along with the production of more whistles, participants unwittingly used a subtle prosodic feature to compete over a resource (ice-cream scoops). Winners can be identified by their propensity to accentuate the first whistles blown when replying to their partner, compared to the following whistles. Naive listeners correctly identified this prosodic feature as a key determinant of which whistler won the interaction. These results suggest that in the absence of other communication channels, individuals spontaneously use a subtle variation of sound accentuation (prosody), instead of merely producing exuberant sounds, to impose themselves in a conflict of interest. We discuss the biological and cultural bases of this ability and their link with verbal communication. Our results highlight the human ability to use non-verbal communication in a negotiation process.

## 1. Introduction

Many animals communicate to reduce the cost of physical confrontation in conflict resolution. The winner of the conflict has priority access to limited resources, theoretically because the way individuals communicate reliably reflects their motivation or their competitive ability [1,2]. Transferring information on the motivation to compete does not necessarily require a complex language. In non-human animals, competitors use conventional stereotyped signals, with highly motivated individuals producing extravagant displays such as intense or numerous conspicuous calls, which indicate the willingness to compete [3].

Conflict of interest takes place whenever the attainment of the goal by one party excludes or limits its attainment by the other [4]. During human conflicts, beside verbal communication, non-verbal features play a substantial role in the inter-individual interaction (e.g. [5]). Many studies highlighted that non-verbal behavioural rules allow individuals to detect situations of conflict (e.g. [6]), but also to assess dominance, motivation or hierarchy status—parameters indicating who holds the power and has the strongest influence during interactions [7,8,9,10]. The way each participant of a social interaction views a conflict of interest is conveyed by non-verbal signals, such as facial expressions, posture, voice intensity and prosody (intonation, tone, stress and rhythm of vocalizations) [11], which change according to status and conflict intensity. For instance, during a conflict, individual variability in speech pitch and loudness increases [10], while speakers interrupt each other more frequently [12]. Observers are thus able to evaluate speakers’ affect and dominance on the basis of content-filtered speeches, using visual and/or vocal cues alone [13,14]. The study of non-verbal signals has allowed the development of automatic tools to identify dominance and related negotiation patterns from audio-visual media (e.g. [15], and finally to predict conflict outcome [16].

Previous works conducted on human non-verbal signals during conflict are mostly based on regular conversations and/or role-play interactions, recorded in the laboratory or extracted from media. In these settings, verbal factors, such as syntax and words’ meaning, covary with non-verbal cues. Hence, it is difficult to disentangle the spontaneous use and perception of non-verbal cues, independently of verbal communication. In the present study, we chose an alternative way to remove the effects induced by visual contacts and speech content (i.e. meaning of words) on the contest process. Using human ability to perform conversation play with simple noise or visual signs [17,18,19], we set up an experiment in which subjects used whistles as a means of communication. This setting permits us to get rid of the possible confounding factors of verbal communication. Because acoustic vocal cues seem particularly important to infer dominance [10], the whistle, which can be blown at different intensity and rhythm, is a suitable tool to study non-verbal communication. We aimed to tackle (1) the spontaneous use of acoustic features to resolve a contest over a resource and thereafter (2) the detection of such acoustic features by naive subjects listening to the recorded acoustic interactions. We predicted that competition would increase whistlers’ activity, in terms of loudness, duration and number of whistles, their propensity to whistle simultaneously with their partner, and would change the prosodic features of whistles, i.e. increase the change/variability in whistles’ loudness and duration along a sequence of whistles [10]. These acoustic features are also expected to determine the winner of a contest over a reward.

In a first experiment, referred to as the “Whistle Dialog Game”, pairs of subjects were asked to communicate using a whistle. We asked players to whistle during periods said to be non-competitive (i.e. there was no conflict of interest, implying that whatever the behaviour of players nobody would obtain a resource) and during periods said to be competitive (i.e. based on the behaviour of players, only one individual would receive a food reward in the form of an ice cream scoop). Contestants could not see each other and could only use whistle sounds, without any previous agreement of any sort on the way to communicate. Pairs of subjects competed for three successive rewards. At the end of each contest, whistlers indicated which one won the reward. The aim of this experiment was twofold. First, we determined to which extent non-verbal whistling communication was influenced by the need to compete, i.e. which acoustic features changed between non-competitive and competitive whistling dialogs. Second, we tested the ability of participants to resolve a conflict using whistle sounds. In particular, we analysed the acoustic characteristics of the dialogs leading to an agreement and the acoustic characteristics of the designated winner.

In a second experiment of playback, listeners, to whom we broadcast competitive interactions between two individuals recorded during the first experiment, were asked to identify the winner. Our aim was to assess whether naive listeners can infer competition success using acoustic cues, and whether it is concordant with the agreement reached by the dialog participants. Finally, we created a synthetic whistle dialog in which we manipulated one single acoustic cue, which was found in the first experiment to be correlated with the likelihood of winning the reward. This synthetic dialog was broadcast to the naive listeners who had to infer who won the contest.

## 2. Methods

### Experiment 1—Whistle Dialog Game

In July 2011, we invited subjects to participate to a communication experiment where two individuals blow a whistle to compete over ice-cream scoops. There were 90 participants representing 45 pairs in total aged on average 26 ± 4 years (all students or employees from the University of Lausanne, Switzerland). 50% of participants were native French-speakers (from Switzerland, France or Belgium) and for them the instructions were given in French. For other groups, in which one or both were non-French speakers (originated from Asia (7), Africa (1), South or North America (7) or Europe (30)), the instructions were in English. Whistlers saw each other before the beginning of the experiment and thus knew with whom they interacted. Most subjects knew their partner (55% were colleagues and 35% were friends or family). However, during the experiment they did not see each other and could only interact by blowing a whistle, as they were separated by a partition and did not talk or laugh.

**Fig 1:**
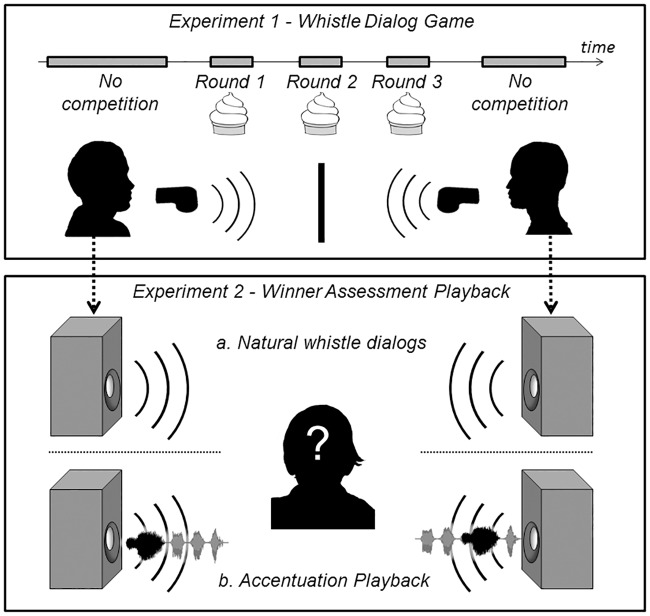
Schema of the experiments. Experiment 1 –Whistle Dialog Game. Experiment 2 –Winner Designation Playback, based on (a) competitive rounds from the experiment 1 and (b) synthetic playback with utterance accentuated either on the first whistle (left) or in the middle of the sequence (right).

The experiment consisted of a first non-competitive interaction lasting 3 min followed by three competitive rounds of 1 min, and a final non-competitive interaction of 2 min (Fig 1). The instructions were given at the beginning of each round. Subjects were not aware of the number and the length of experimental rounds so that this information did not influence their behaviour [20]. During non-competitive interactions, whistler pairs were asked to interact freely by whistling and we told them that the outcome of this non-competitive interaction had no influence on which of the two would get ice cream scoops later on. During each round of competition, they were asked to communicate in order to settle who would receive an ice cream reward (after each competitive round one person can win an ice cream scoop and hence participants can win up to three ice cream scoops). At the end of each competitive round, indicated by the experimenter, the person who assumed s/he had won had to indicate it by blowing the whistle. The first subject to whistle obtained one reward (which was given at the end of the experiment). If both subjects, or none of them, whistled at the end of a competitive round, the competition was considered “unresolved” and nobody obtained the reward. At the end of the whole experiment, the subjects were asked to write down whether rewards were fairly distributed or not. Contestants reached an “agreement” during a round if the two players approved the reward attribution, i.e. one partner reclaimed the reward by whistling (the “winner”) and his/her partner indicated that the reward distribution was fair.

Whistles were plastic toys renewed for each subject and placed at a fixed distance from a microphone (Microspot AR-666). The microphone of each participant was acoustically isolated in a tube, so that it did not record the whistles of the other participant. We automatically extracted the different components of the whistles from the recordings using Avisoft-SASlab Pro’s pulse train analysis (Avisoft Bioacoustics, Glienicke, Germany).

Participants whistled one after the other, one “utterance” defined as a sequence of whistles without partner’s interruption. We considered the following simple acoustic variables for the analyses: (1) mean whistle loudness (mV), (2) mean whistle duration (s), (3) total number of whistles, (4) pause intervals (s) between two successive whistles from the two participants (hereafter referred to as turn-taking gap) and (5) the proportion of overlapping whistles (number of whistles by a given player which started before the end of the partner’s whistle, over the total number whistles emitted by this player). We noticed that the first whistle of an utterance was usually more accentuated than the others, as it was significantly longer and louder (first vs. following whistles’ duration: 0.35±0.03 vs. 0.24±0.02 s, S = 1867, P < 0.0001; first vs. following whistles’ loudness: 0.13±0.01 vs. 0.11±0.01 mV, S = 1438, P < 0.0001). In order to study the prosody pattern used by whistlers and its variation, we hence analysed whistle blow accentuation (in duration and loudness) within an utterance. (6) Duration accentuation and (7) loudness accentuation were measured as the mean difference over all utterances between the first whistle of an utterance and the following whistles of the utterances, in duration (s) and loudness (mV), respectively. These prosodic traits were correlated with the coefficient of variation of whistle duration and loudness, respectively (Spearman correlation: r_423_ = 0.31, P > 0.0001, r_423_ = 0.27, P > 0.0001, with one line per individual and per round).

### Experiment 2 –Winner Designation Playback

In May 2015, we invited 30 subjects aged on average 23 ± 1 years to listen to four successive whistle dialogs. Three of these dialogs (Experiment 2a) were from competitive rounds recorded in Experiment 1 and one was a synthetic playback dialog (Experiment 2b) (Fig 1). The synthetic playback dialog was broadcast in 3^rd^ position, but listeners were not aware that one of the dialogs was synthetic. Listeners were asked to indicate who won the competition at the end of an exchange between two persons competing for a resource using a whistle (Question form in S1 File). No further instructions were given. Listeners were asked to add whether they were “sure”, “not totally sure” or “not sure” about who won the interaction. Participants were students or assistants of the Science Faculty of the University of Bern (Switzerland) and did not know the protocol of the Experiment 1; 22 were native German- or Swiss-German-speakers, 2 from Asia, 1 from America or 5 from other parts of Europe than Germany or Switzerland. Instructions were given in English.

#### Experiment 2a. Natural Whistle Dialogs

We tested whether third-party listeners detected the winner of a “Whistle Dialog Game” (Exp. 1), when listening to whistle dialogs differing only in prosodic features. We hence selected competitive exchanges from the Whistle Dialog Game performed in 2011, with the criteria that the contestants reached an agreement and that the two contestants differed in less than 10% in the total number of whistles and in average whistle duration. We found 33 competitive rounds following these criteria and we randomly picked 11 of them to be broadcast. In these exchanges, the winners produced 48±2% of total whistles and the loser 52±2% on average (Wilcoxon signed rank test: *P* = 0.52), winners blew in the whistle for on average 0.17±0.02 s and the loser during 0.16±0.02 s (*P* = 0.32). The winners overlapped the whistles of their partner on 4.8±1.1% of the occasions and the loser on 3.7±1.0% (*P* = 0.57). Whistle loudness was on average slightly higher for winners than losers (0.08±0.01 vs. 0.05±0.01 mV, *P* = 0.01), and because we were interested in whether prosody can be used to identify the winner of a competition, we equalized the whistle loudness of both players using Audacity freeware (http://audacity.sourceforge.net).

#### Experiment 2b. Accentuation Playback

We tested whether listeners perceived the prosodic feature found to predict the outcome of a Whistle Dialog Game (the “duration accentuation”, see Results). To this end, we created synthetic dialogs in which two whistler contestants differed only on this prosodic feature. We generated playbacks for which one whistler accentuated the first whistle of each utterance (high duration accentuation, i.e. the first whistle of each utterance was the longest), while for the other whistler, the longest whistle was in the middle of the utterance (low duration accentuation, Fig 1). Apart from this difference in duration accentuation, the two playback whistlers produced exactly the same 10 utterances one after the other, in a randomly assigned order. We built five different playbacks, by selecting 10 natural utterance sequences (containing 3 to 8 whistles) from five individuals of the Experiment 1. We modified the order of the whistles in the selected 10 utterances using Audacity software, so that each utterance had a version in which the longest whistle was at the beginning (for one whistler) and one version in which the longest whistle was in the middle (for the other whistler). The whistler ending the playback dialog was randomly assigned at each experiment.

Each whistler was emitted on a different loudspeaker (BeoPlay A2, Bang & Olufsen, Denmark), allowing listeners to easily distinguish the two players. The loudspeakers’ side (left or right) was randomly assigned at each experiment.

### Statistical Analyses

#### Effect of competition on Whistle Dialog Game (Exp. 1)

In order to test whether individuals whistled differently during the competitive and non-competitive dialogs, we averaged the acoustic features of each of the three competitive rounds and of the two non-competitive interactions for each whistler in the Whistle Dialog Game. For each pair of individuals, we then separately averaged the six means for competitive rounds and four means for non-competitive interactions. We compared the mean final values of the competitive and non-competitive whistle dialogs using Wilcoxon signed rank tests (paired difference tests).

#### Achieving an agreement in the Whistle Dialog Game (Exp. 1)

To analyse which acoustic features were related to the likelihood of reaching an agreement during the competitive rounds of the Whistle Dialog Game, we defined agreement/disagreement in each round as the dependent variable, in a generalized linear mixed model (GLMM) with binomial distribution (d.f. = 1,56). Because each pair competed during three rounds, we set pair as random factor. As independent terms, we set the absolute difference between the two partners for the different acoustic features, round number (1^st^, 2^nd^ or 3^rd^) and the interactions between round number and other independent terms.

Some intrinsic factors related to the pair may have influenced the likelihood of reaching an agreement (e.g. if they were acquainted, or if they relied on a hierarchy status or personality perceived before the experiment). To control that acoustic communication *per se* influenced the outcome of the contest, we analysed the within pair change in acoustic features according to whether partners found an agreement or not (for the 28 pairs which did not reach an agreement during all the rounds). We separately averaged the acoustic features of rounds leading to an agreement and rounds leading to a disagreement and compared the values with Wilcoxon signed rank test.

#### Winning success in the Whistle Dialog Game (Exp. 1)

In order to analyse which factors predicted who won the reward in the Whistle Dialog Game, we compared the acoustic features of winners and losers during resolved competitive rounds (in which only one whistler claimed for the reward at the end of the round). As dependent variable, we set the contest outcome (winner or loser of a round) of a randomly chosen individual of each pair in a GLMM with binomial distribution. As independent variables, we set the mean differences between the two whistlers in the acoustic features, round number (1^st^, 2^nd^ or 3^rd^) and the interactions between round number and other independent terms. Pair was set as random factor. The results of the simple effects of GLMMs are given from models in which the interactions are removed (as they appeared to be non-significant, see results). The covariates were not collinear (all r < 0.4 in Pearson correlations).

As for agreement achievement, some intrinsic factors related to the pair may have influenced the likelihood of winning (e.g. if pairs relied on an assessment of dominance performed before the beginning of the experiment). We thus compared the change of whistling behaviour within an individual, during the three successive rounds, according to whether s/he was the loser or the winner (N = 36 individuals, who were alternatively winner and loser).

#### Success of Winner Designation (Exp. 2.a)

To determine whether the listeners guessed the identity of the winner of the Whistle Dialog Game, we performed GLMM (with logit link), with designation response (1 for correct / 0 for incorrect designation of winner) as dependent binomial variable. Because the 30 subjects heard 3 different dialogs, listener identity was set as random factor, as well as the playback dialog (in total 11 different dialogs were broadcast). The test of intercept effect in an empty model indicated whether the responses were significantly different from the random expectation of 50%.

#### Effect of prosody on Winner Designation (Exp. 2.b)

In the artificial playback dialog, we examined whether the whistler with high duration accentuation (i.e. accentuation of the first whistle of utterances) or the other one (low duration accentuation, i.e. accentuation in the middle of utterances) was selected as winner by listeners, using a binomial test.

### Ethics Statement

Before starting the first experiment, subjects signed a free consent form. All participants were over the age of legal majority. The first experiment was approved by the Human Research Ethics Committee (Vaud canton). The second experiment being a questionnaire, it does not fall within the competences of the Committee. Data were anonymised for both experiments and no identifying participant information was collected.

## 3. Results

### Experiment 1—Effect of competition on Whistle Dialog Game

When competing for a resource in the form of an ice-cream scoop, participants produced louder sounds, longer whistles and emitted their whistles quicker after their partner than during the pre- and post-competition phases (Table 1a). Whistlers did not produce significantly more whistles per minute (Table 1a) and did not overlap significantly more during competitive rounds than non-competing interactions.

**Table 1:** (a) Effect of conflict on whistle dialog. (b) Relation between the likelihood of winning a contest and whistlers’ behaviour.

	a. Conflict effect				b. Conflict outcome			
	Comparison between competitive and non-competitive interactions				Comparison between winners and losers of competitive rounds			
	Competition	Non-competition	*S*	*P*	Winner	Loser	*F*	*P*
**Loudness (mV)**	**0.13±0.01**	**0.10±0.01**	**-234**	**0.0035**	0.13±0.1	0.12±0.1	1.43	0.24
**Duration (s)**	**0.36±0.05**	**0.25±0.01**	**-298**	**0.0002**	0.29±0.3	0.33±0.7	0.58	0.45
**Turn-taking gap (s)**	**1.4±0.1**	**1.8±0.1**	**204**	**0.016**	1.42±0.13	1.68±0.16	0.54	0.47
**Number of whistles**	37±2	36±2	30	0.73	**41±3**	**36±2**	**7.95**	**0.007**
**Overlap (%)**	4.2±0.6	3.1±0.4	-92	0.20	3.8±0.6	3.9±0.6	0.05	0.82
**Duration accentuation (s)**	0.11±0.02	0.10±0.01	-12	0.88	**0.14±0.02**	**0.07±0.02**	**10.18**	**0.004**
**Loudness accentuation (mV)**	0.05±0.01	0.05±0.02	49	0.17	**0.03±0.01**	**0.04±0.01**	**4.06**	**0.049**

Accentuation was estimated as the mean difference between the first whistle of an utterance and the mean of the following whistles, in duration or loudness. (a) Wilcoxon signed rank tests compared the difference in mean acoustic variables between competitive and non-competitive situations in pairs of whistlers. (b) A generalized linear mixed model evaluated the effects of individual acoustic variables on the likelihood of winning a contest.

Accentuation was not significantly different between competitive and non-competitive rounds (Table 1a).

### Experiment 1—Achieving an agreement in the Whistle Dialog Game

In 88% of all 135 competitive rounds, only one individual of the whistling pair claimed for the resource and in turn received an ice cream scoop. This settlement of the contest was considered “fair” by his/her partner in 82% of cases. Hence, in 72% of all rounds (82% of 88%), pairs reached an agreement over ice cream distribution. 12% of the rounds remained unresolved, 7% because both partners claimed the food and 5% because none of them claimed it.

A competitive round was more likely to lead to an agreement when the difference in utterance accentuation between the two partners was higher. In other words, when one individual accentuated the first whistle of utterances to a larger extent than his/her partner, they both agreed about which one should obtain the reward (absolute difference between partners in duration accentuation when competition lead to agreement *vs*. disagreement: 0.27±0.02 *vs*. 0.17±0.01 s; *F*_1,71_ = 9.40, *P* = 0.003). No other acoustic features predicted the agreement success (absolute difference in loudness: *F*_1,66_ = 0.09, *P* = 0.77; absolute difference in duration: *F*_1,66_ = 0.18, *P* = 0.68; absolute difference in turn-taking gap: *F*_1,66_ = 0.03, *P* = 0.85; absolute difference in number of whistles: *F*_1,66_ = 0.19, *P* = 0.67; absolute difference proportion of overlapping whistles: *F*_1,66_ = 0.16, *P* = 0.69). The order of competitive round (1^st^, 2^nd^ or 3^rd^) was not significant, nor the interaction between round and acoustic features (all *P* > 0.05). Pairs of competitors which did not always reach an agreement during competitive rounds were more different in duration accentuation when they reached an agreement than when they did not (S = 90, *P* = 0.038).

### Experiment 1—Winning success in the Whistle Dialog Game

**Fig 2:**
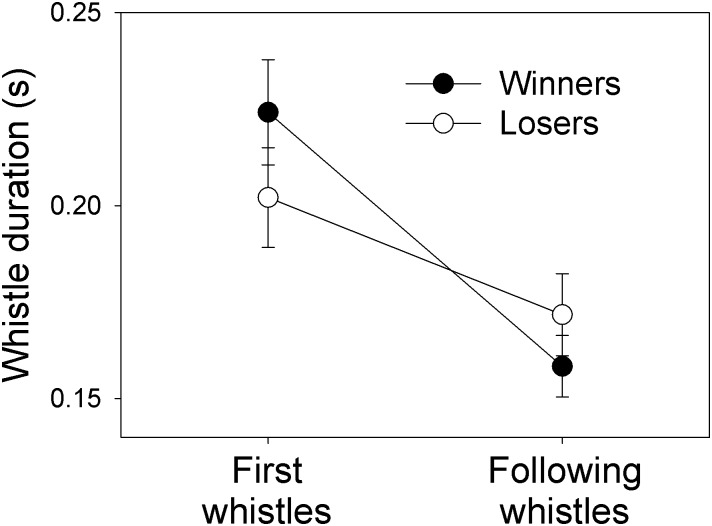
Difference in prosody between winners and losers. Duration of the first and following whistles of utterances, for winners and losers who reached an agreement in whistle contests.

In competitive rounds that were resolved and hence for which the resource was distributed, the winner accentuated the first whistle of utterances to a larger extent than losers (Fig 2; effect of accentuation difference between partners on winning probability: *F*_1,56_ = 10.18, *P* = 0.004; Table 1b). Furthermore, the winners produced more whistles than their partner (effect of difference in number of whistles between partners on winning probability: *F*_1,56_ = 7.95, *P* = 0.007; Table 1b). The differences between partners in whistle loudness, whistle duration, turn-taking gap or proportion of overlapping whistles did not predict the success of a whistler (Table 1b). The order of competitive round (1^st^, 2^nd^ or 3^rd^) was not significant, nor the interaction between round and acoustic features (all P > 0.05). The sex of the whistler did not influence his/her propensity to win the reward (*F*_1,56_ = 0.07, *P* = 0.79).

In the 36 pairs where the winner was not always the same individual across the three rounds, the accentuation difference changed significantly between rounds where one or the other individual won (Wilcoxon signed rank test: S = 133, *P* = 0.034), as well as the difference in number of whistles (S = 187.5, *P* = 0.003).

### Experiment 2—Winner Designation

#### Success of Winner Designation (Exp. 2.a)

**Fig 3:**
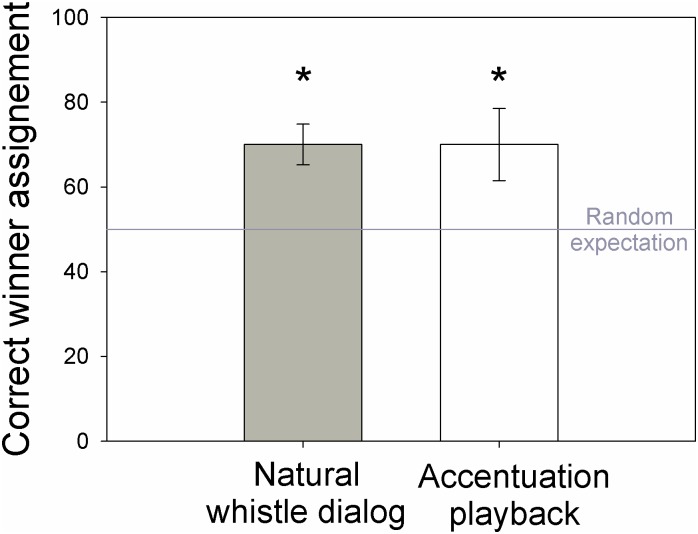
Designation of winner by naïve listeners. Proportion of time listeners designated the winner consistently with the players of the Whistle Dialog Game (left). Proportion of time listeners designated as winner the playback emitting the long whistles at the beginning of utterances rather than in the middle of utterances (right).

70% of the naive listeners designated the winner concordantly with the participants of the Whistle Dialog Game, which is significantly different from random (Fig 3; GLMM: t = 4.24, *P* < 0.0001). The evaluation success was similar for the 3 dialogs (1^st^ natural whistle dialog broadcast: 71% of success, 2^nd^: 68%, 3^rd^: 68%).

#### Effect of prosody on Winner Designation (Exp. 2.b)

70% of listeners designated the whistler that accentuated the first whistle of each utterance as winner (significantly different from random, unilateral binomial test: Z = 2.19, P = 0.014).

For 16% of replies, listeners were “not sure” while in 40% of replies listeners were certain of their choice. However, the proportion of correct (or expected) choices did not differ significantly between people who said that they were sure or not sure of their response (70% *vs*. 68%).

## 4. Discussion

Inter-individual communication can use other channels than verbal speech. Without any previous training, most participants were able to resolve a contest and reach an agreement solely by emitting sounds with a whistle. The effectiveness of this mode of communication is further demonstrated by the observation that naïve third-party listeners correctly identified the winner of a contest taking place between two subjects. Indeed, we found a consistency between how whistlers attributed the rewards of the contest and how listeners identified the winner, although naïve third-party listened to dialogs for which the winner and loser emitted a similar number of whistles and of similar intensity. In conclusion, our study has demonstrated that individuals can spontaneously use non-verbal acoustic signals to negotiate simple intentions in a conflictual situation. Our results also highlight the importance of non-verbal communication in negotiation process and the ability of people to use non-verbal cues to convince an opponent.

Our results confirm that non-verbal behaviour is a source of information on many aspects of inter-personal relationships [11]. Speaker’s affective state, such as nervousness or confidence, can indeed be perceived by non-verbal expressions [13,21]. The sensitivity to prosodic cues in particular appears early in life, even preverbal infants discern such cues in speech processing [22,23]. The prosody of speech highly influences how people perceive the speaker, although seemingly without being aware of it [24]. This is consistent with the fact that in our setting, listeners were often unaware of their ability to detect the winner of Whistle Dialog Games, as they were uncertain of their reply in 16% of trials, but still assessed the correct winner in 68% of these replies. Listeners could not justify their choice and were not able to describe the difference between whistlers in terms of acoustic features. In their comments after the experiment, they claimed having chosen the “more aggressive” or “more persuasive” contestant (A.D., personal observation). The confidence of individuals in their social judgment is generally not a good predictor in their actual ability, for instance when judging deception or assessing the relationship between unknown persons [25,26]. The lack of relationship between confidence and accuracy may be due to the fact that they are differently influenced by factors such as self-confidence, sex or age [26].

Two types of sound characteristics were modulated in Dialogs Games. The first type was related to “performance”. Individuals increased the duration, rhythm and loudness of whistles when competing and produced numerous whistles to persuade their partner and win the contest. Interestingly, contestants used a second type of sound characteristics, the accentuation of the first whistle of utterances, which can be related to “prosody”. Whistlers used this subtle sound variation to affirm their dominance or motivation to win the contest. When they took the turn, winners produced a longer initial whistle than losers. In contrast, lengthening whistles within an utterance did not affect the winning success. Listeners hearing two contestants differing only in this accentuation evaluated that the winner was the one lengthening the first whistle. Competition for resources can hence be solved through a prosodic feature only, recognised as a dominant cue by listeners.

In situations where verbal communication is impossible, people are known to improvise gestures [27] or vocal symbols [19]. This ability underlines human potential to create non-verbal signals to communicate various meanings and emotions. In our experiment, the fact that the sound characteristics used to win the Dialog Game were recognized by naïve listeners, suggests that these sound characteristics have a *biological* or *cultural* basis. The ability to use and understand “performance” and “prosodic” acoustic cues independently of verbal communication are likely to be related to different phenomenon. We hypothesise that “performance” acoustic features have biological basis, while the “prosodic” feature has a cultural basis. Indeed, communication during conflict may require “performance” signals, i.e. conspicuous and costly displays to influence the behaviour of conspecifics. Theoretically, this is due to only competitive or highly motivated individuals being willing to pay the cost of conspicuous displays [28,29]. Non-human animal communication and human language would share this common biological characteristic when individuals display their dominance during conflicts, as shown by human ability to detect the social meaning of male dominance vocalizations of a macaque monkey [30], although this ability might be related to experience-dependent cognitive mechanisms [31]. In the Whistle Dialog Game, whistlers produced longer, louder and more rapid whistles when competing. In the same vein, speakers engaged in a dispute usually talk loudly [32] and with a high rate of interruption [33]. In our experiment, whistlers produced more whistles to maximise their access to a reward. Vocal activity in speech, i.e. the proportion of time an individual is speaking, is often associated with dominance or hierarchy status [9,34]. Hence, although human communication is of totally different nature than non-human animal communication in many aspects, human competitors may follow fundamental rules shared by many animal signalling systems when they manipulate sounds to compete, that is to say the use of performance signals.

On the other hand, beside the number of emitted whistles (and contrary to most animal signals), whistlers used and perceived a prosodic cue, the accentuation of the first whistle of utterance, when contesting during Dialog Games. This signal is not a performance signal, as the accentuation of sound was not higher but differently distributed in winners than losers. The reason why this signal was used and understood by a majority of contestants can only be speculated. It might be related to whistlers mirroring prosodic features used during verbal interactions. Whistle blowing cannot be directly compared with voice production, however, whistlers might have mimicked speech, the most commonly used auditory signal. Adults indeed interpret/classify the variation of simple tone loudness and pitch according to the position of word stress in their native language [35]. Syllable accentuation or lengthening of some parts of an utterance is observed in various languages [36] and even in baby babbling [37]. However, although dominance is known to influence the variability of speech vocal pitch and amplitude [10], the relation between dominance and the specific stress of some part of a sentence has not been studied so far, as far as we know. Moreover, the tendency to lengthen the initial or the final part of utterances varies across languages [36,37]. The first experiment was mainly performed with French-speakers, but with a high variability in sample, and the listeners of second experiment were mostly German-speakers, which did not prevent them from detecting the contest outcome. The repeatability of our results suggests that the use of this prosodic feature—the accentuation of the first whistle—was independent of subjects’ mother tongue. Our experiment has hence provided evidence that a prosodic feature can be used spontaneously to solve a contest. The cultural or biological origin of this prosodic feature is hence to be studied. The next step would be to return to natural speech and test whether it is used in verbal speech and in which competitive and cultural context. Our work should trigger further studies to determine whether accentuation in the beginning of utterance is used in verbal negotiation, and to what extend it can help to predict negotiation outcome.

## Supporting Information

S1 File Question form given to subjects of Experiment 2. (PDF)
