# Supplementary material for: Prosody Predicts Contest Outcome in Non-Verbal Dialogs
Source: PLoS One. 2016 Dec 1;11(12):e0166953. doi: 10.1371/journal.pone.0166953 (PMC5132166; doi:10.1371/journal.pone.0166953)
Supplement: S1 File — (PDF) [file pone.0166953.s001.pdf]

Supporting information

Question form given to subjects of Experiment 2

Listen carefully to the recording.
Two persons are competing for a resource using a whistle.
After about 1 min, the competitive dialog is interrupted.
Indicate **who** won the competition **according to you**.

① ☐ ← Left Right → ☐

Certainty of your answer:
- I answered by chance: ☐
- I am not totally sure: ☐
- I am quite sure of my answer: ☐

---

② ☐ ← Left Right → ☐

Certainty of your answer:
- I answered by chance: ☐
- I am not totally sure: ☐
- I am quite sure of my answer: ☐

---

③ ☐ ← Left Right → ☐

Certainty of your answer:
- I answered by chance: ☐
- I am not totally sure: ☐
- I am quite sure of my answer: ☐

---

④ ☐ ← Left Right → ☐

Certainty of your answer:
- I answered by chance: ☐
- I am not totally sure: ☐
- I am quite sure of my answer: ☐

Individual n° _______ Date _______ Hour _______

To be filled at the end:

Did you enjoy the experiment?

Yes	Rather Yes	Rather No	No
☐	☐	☐	☐

Age:
Sex: ☐ Male<br>☐ Female
Mother tongue:

Individual n° _______ Date _______ Hour _______
